# Reduced social preferences in autism: evidence from charitable donations

**DOI:** 10.1186/1866-1955-4-8

**Published:** 2012-05-17

**Authors:** Alice Lin, Karin Tsai, Antonio Rangel, Ralph Adolphs

**Affiliations:** 1 California Institute of Technology, Pasadena, CA, USA; 2 Princeton University, Princeton, NJ, USA; 3 HSS 228-77, Caltech, Pasadena, CA, 91125, USA

## Abstract

**Background:**

People with autism have abnormal preferences, ranging from an apparent lack of preference for social stimuli to unusually strong preferences for restricted sets of highly idiosyncratic stimuli. Yet the profile of preferences across social and nonsocial domains has not been mapped out in detail, and the processes responsible remain poorly understood.

**Methods:**

To assess preferences across a range of stimuli, we measured real monetary donations to 50 charities spanning categories pertaining to people, mental health, animals, or the environment. We compared the donations made by 16 high-functioning adults with autism to those made by neurotypical controls matched on age, gender and education. We additionally collected ratings of how people evaluated the different charities.

**Results:**

Compared with controls, high-functioning adults with autism donated less overall and also showed a significantly disproportionate reduction in donations to people charities compared with donations to the other charities. Furthermore, whereas controls discriminated strongly between different people charities, choosing to donate a lot of money to some and very little to others, much less discrimination was seen in the autism group. Ratings that probed how participants constructed their preferences did not differ between groups, except for a difference in the perceived impact of pictures and text information about people charities. Strikingly, there were some charities related to mental health, and autism in particular, to which the autism group donated considerably more than did the controls.

**Conclusions:**

People with autism were found to have reduced preference and sensitivity towards charities benefiting other people. The findings provide evidence for a domain-specific impairment in social cognition in autism spectrum disorder, and in particular in linking otherwise intact social knowledge to the construction of value signals on which preferences regarding other people are based.

## Background

People with autism spectrum disorder (ASD) show behaviors suggesting abnormal preferences for stimuli. For instance, certain sensory stimuli or unfamiliar situations appear to be highly aversive, whereas other stimuli and familiar or repetitive situations appear to be desired; often, idiosyncratic objects can elicit abnormal attention and interest [1,2]. Together with these sometimes exaggerated preferences restricted to a specific set of unusual stimuli, there is a reduction in preferences for other people [2,3]. These findings have motivated the hypothesis that ASD involves a domain-specific impairment for the valuation of social stimuli [4,5]. Nevertheless, the extent to which these impairments in ASD are confined to the domain of social processing remains an open question.

In this study, we addressed this open question by investigating how the preferences of participants with ASD compare to those of matched controls in a real charitable donation task. We chose a large number (N = 25) of charities benefitting people (for example, American Red Cross), but also nine charities that would benefit mental health (for example, Autism Research Institute), ten charities benefitting animals (for example, African Wildlife Foundation), and six charities benefitting the environment (for example, Heal the Bay). Participants were given pictorial and descriptive information about each charity, asked to choose an amount to donate to that charity over the internet, and asked to rate each charity on a number of attributes. The charitable task is an interesting framework with which to address this issue because it involves the valuation of complex stimuli in more naturalistic behavioral settings than those used in previous experiments [6-8].

With respect to the stage of processing, impairments might arise from motivational, attentional, sensory, or more complex cognitive processing abnormalities. One theory consistent with observations in ASD and with our current framework from cognitive neuroscience for understanding reward learning is that a lack of motivational and attentional impairments for social stimuli early in development could result in later impairments in perceptual and cognitive processing of social stimuli that might depend on normal social input during development [3,9,10]. For instance, it is known that neural and behavioral specializations for face processing depend in part on expertise with faces traceable to early domain-specific processing, and so one plausible scenario could be that an early lack of motivation to orient towards faces results in reduced sensory input about faces and later reduced ability to process faces. In support of a developmental role for such altered preferences, it is known that children with autism fail to orient normally to social stimuli [3], and one study found that a person with autism showed activation of the fusiform face area not to real faces, but faces of preferred cartoon characters [11].

Whereas basic preferences for stimuli can be investigated using measures such as eye-tracking in infants, it is more difficult to assess complex real-world preferences in adults. We wanted to capture possible impairments at any stage of processing during complex decisions based on relative preferences, and thus chose to measure anonymous charitable donations involving real money. While charitable donations no doubt are based on preferences for the charities, the online computation of such preferences likely draws on multiple processes ranging from empathic and altruistic considerations as well as reward processing. A recent functional magnetic resonance imaging study [12] demonstrated that charitable donations activate regions within the ventromedial prefrontal cortex thought to encode a common reward currency [6,7], as well as regions in the insula and superior temporal sulcus likely involved in empathy, social attention and altruistic thinking [13,14]. A model motivated by this and related studies is that social preferences during charitable giving are constructed via inputs to the ventromedial prefrontal cortex from regions concerned with processing information about the benefits to others [12], just like this region of the prefrontal cortex constructs values from sensory representations in more posterior cortices in general [15].

Several of these putative processing stages are thought to be impaired in people with autism: both basic social reward processing and more complex evaluations of social stimuli that depend on context, mentalizing or empathy, have been reported to be abnormal in autism (see Discussion for further details). Our primary goal in the present study was not to dissect these processing components, but rather to provide an inventory across different types of stimuli, some social and others not.

## Methods

### Participants

We recruited 16 high-functioning adults with a Diagnostic and Statistical Manual, Fourth Edition diagnosis of autism or Asperger’s syndrome (four female) and 16 age- and education-matched controls (three female; see Table 1 for details). All participants with ASD met cutoff scores for autism or Asperger syndrome on the Autism Diagnostic Observation Schedule (ADOS) Module 4 [16], and 13 out of 13 participants assessed also met criteria on the Autism Diagnostic Interview-Revised [17]. All participants had an intelligence quotient (IQ) in the normal range as assessed with the Wechsler Adult Intelligence Scale [18] and gave informed consent to participate in the studies under a protocol approved by the Institutional Review Board of the California Institute of Technology.

**Table 1:** Summary of demographic and background information about the participants

	**n**	**Gender**	**Age**	**Full-scale IQ**^**a**^	**Education (years)**	**IRI**^**b**^**(EC + PT)**
With ASD	16	12 males 4 females	31.4 (12.3) [19-57]	110 (12.7) [93-133]	15.8 (2.1) [9-18]	24 (11.7) [6-42]
Matched controls	16	13 males 3 females	31.1 (12.7) [19-56]	114 (13.6) [94-133]	16.1 (1.4) [13-18]	37 (5.4) [27-43]
	**ADI**	**ADOS**	**SRS**			
With ASD	45 (10.5)[27-61]	17 (5.7)[11-25]	91 (26) [43-126]			

^a^The full-scale IQ from the Wechsler Adult Intelligence Test [18]; ^b^IRI is the sum of the empathic concern and perspective taking sub-scores from the Davis interpersonal reactivity index [19]. ADI: Autism Diagnostic Interview; ADOS: Autism Diagnostic Observation Schedule; ASD: autism spectrum disorder; SRS: Social Responsiveness Scale. Data are presented as the mean with the standard error in parentheses and the range in brackets.

### Experimental tasks

Participants took part in the following three sessions in fixed order.

In the first session, they were familiarized with all the charities through a series of simple tasks. First, they were asked to indicate how familiar they were with the charity name. Next, participants were presented with a picture and asked to read a description of the charity’s mission. Last, they were asked to place the charity in the best category among the following choices: environment, animal, people and mental health. While participants were encouraged to provide single assignments, dual categories were allowed in exceptions (for example, charities like Canine Assistants that benefited both animal and people).

Participants’ classifications were not used to derive the assignment of charities to categories used in our analyses, but rather as a check to our pre-assigned categorizations. Participant classifications were nearly identical to ours across all categories and none of the results presented below differ significantly if we use participant categorization of the charities. We assigned charity categories by using a filtering method. If the charities included mention of animals, the environment or mental illness, they were classified in their respective category; otherwise they were labeled a people charity. The list of charities used and their categorizations are presented in Additional file 1 Table S1.

**Figure 1:**
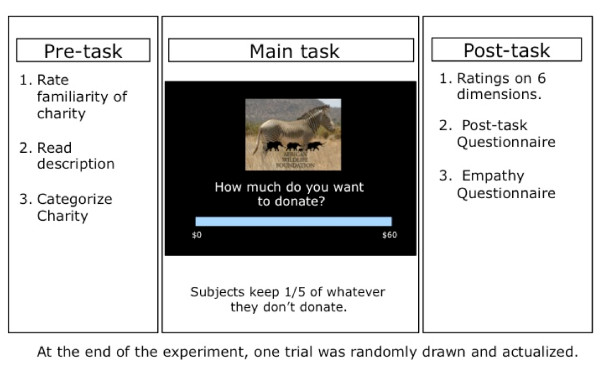
**Schematic of the donation task.** Participants carried out three sessions: first, they were presented with a picture and description of the charity in question, then they decided on their donation (one charity at a time), and finally they provided evaluations of the charity descriptions and pictures through explicit ratings.

In the second session, participants performed the charitable donation task. On every trial, the participant chose how much of $60 they wanted to donate to the charity presented (Figure 1). Participants kept 20 % of whatever amount they chose not to donate. Participants made donation choices for all 50 charities, one at a time, in randomized order. They were told that at the end of the experiment one of their actual choice trials would be randomly selected and implemented, at which point an actual donation was made to the selected charity and they kept any remaining cash. Note that because only one trial was selected to count, the participants could treat each decision as being the only decision made, and did not have to worry about spreading their money across the different charities.

In the third and final session after the donation task, participants rated questions that measured how much the charity would benefit them (for example, ‘how much do you think a $500,000 donation to this charity would help you personally?’), close friends and family, other people, and the world. They also rated the impact of the picture and descriptions they had been given for each of the 50 charities in terms of how effective they felt the charities were in promoting donations (‘to what extent does the charity picture/description increase your willingness to give?’). This session thus provided us with an inventory of explicit knowledge about and evaluations of the charities. The complete set of questions asked is provided in Appendix 1.

After completing the above sessions, participants also completed the Interpersonal Reactivity Index (IRI) [19] personality questionnaire, which measures an individual’s dispositional empathy, a post-task questionnaire that collected demographic background information, and free-response questions about their motivations to give.

All statistical tests were two-tailed unless stated otherwise.

## Results

**Figure 2:**
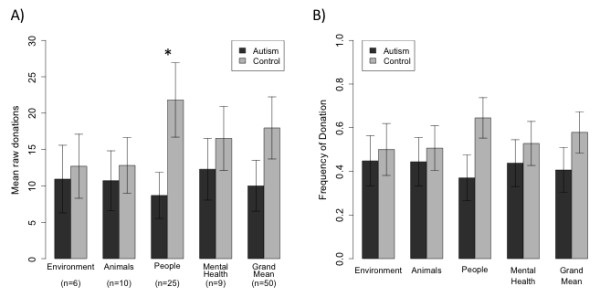
**Mean and frequency of donations across all four categories. (A) Raw donations (mean and standard error of the mean (SEM); not normalized), for the four charity categories, as well as across all charities (Grand Mean).(B)** Probability of donating to a charity in a particular category, means and SEM. Shown is the probability of making any donation, regardless of its magnitude. *P < 0.05.

We first tested the hypothesis that the group of participants with ASD would donate less to people charities. Compared to the control group, the group with ASD donated less often to people charities (37 % versus 65 %; t(30) = −1.97, *P* <0.03, one-tailed) and their mean donations to people charities were lower ($8.69 versus $21.82; t(25) = −2.18, *P* <0.02, one-tailed), but so was the frequency of donations and mean donations to all charities on average, although this effect did not reach significance ($10.01 versus $17.97; t(29) = −1.44, *P* <0.16, Figure 2a,b). Even when excluding any zero donations to a charity, mean donations across all charities from the group with ASD were lower, although again this group difference was not significant ($17.04 versus $28.11, t(23) = 1.89, *P* = 0.07).

**Figure 3:**
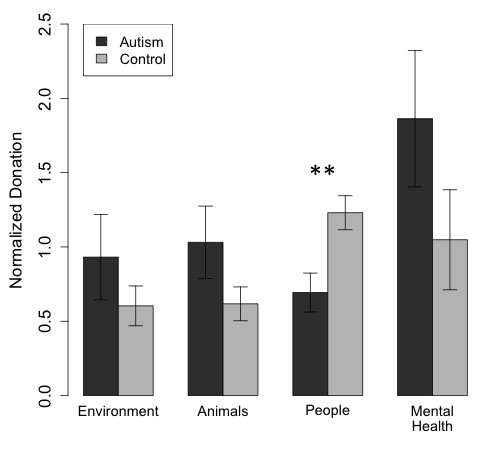
**Normalized mean donations (mean and standard error of the mean), shown for the four charity categories.** Donation amounts were divided for each participant by that participant’s mean donation across all charities. This revealed a disproportionately lower amount donated to people charities than to any other category of charity. **P < 0.01.

To account better for differences in mean donations between individuals within a group, we normalized each participant’s donation by the mean number of dollars he or she donated in the experiment. This revealed a specific abnormality in mean normalized donations specific to the people charities (Figure 3; t(28) = −3.10, *P* <0.002; all other charity categories not significant). A similar result was obtained for median donations per category (t(24) = −2.34, *P* <0.02).

While our hypothesis specifically concerned social preferences, we also carried out a confirmatory mixed analysis of variance (ANOVA) with two levels of group (ASD, control) and two levels of charity category (people, other). This revealed a significant interaction between group and category (F(1,1) = 8.3094, *P* <0.005) and no significant main effects of category or group. Post-hoc t-tests showed that this result was driven by the significant difference between ASD and controls normalized donations to people charities mentioned above. We verified these results with a resampling permutation test. We generated 10,000 random permutation samples and found that fewer than 2 % of resampled differences in mean donation to people charities were higher than what was observed in our data set. In contrast, none of the other charity categories were close to statistical significance (environment: *P* <0.39, animal: *P* < 0.36, mental health: *P* < 0.25; one-tailed).

**Figure 4:**
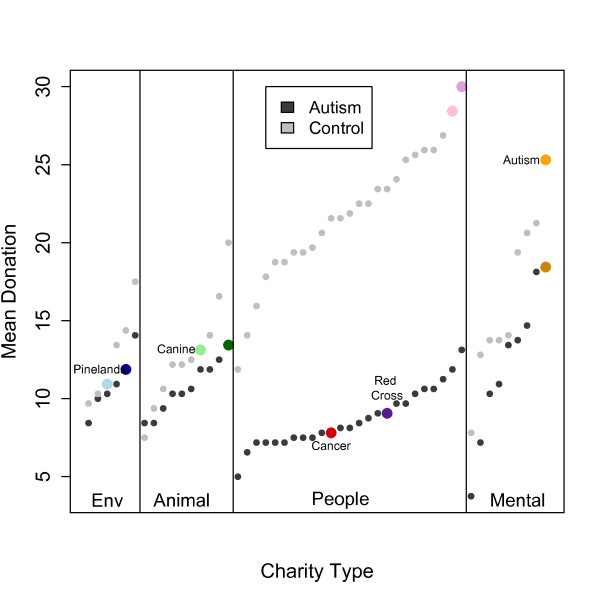
**Mean donations to individual charities, rank-ordered by the donations given by each participant group.** Charities indicated by colored data points correspond to those where the group with autism spectrum disorders showed particularly large differences in their donations compared with donations from those in the control group. Donations from those with autism spectrum disorders are indicated in solid colors and donations from the control group in fainter colors. Pinelands: Pinelands Preservation Alliance (environmental charity); Canine: Canine Assistants (animal charity); Cancer: National Childhood Cancer Foundation (people charity); Red Cross: American Red Cross (people charity); Autism: Autism Research Institute (mental health charity).

We next examined individual charities, rank-ordering them by the mean donations within each category separately for each group (Figure 4). This analysis showed two components to the abnormal donations from the group with ASD. First, it confirmed that the group with ASD donated disproportionately less to the people charities. Second, it revealed a lack of discrimination amongst the people charities: whereas both the ASD and control groups showed a similar spread in donations across individual charities within each category, this was notably absent for the group with ASD in the case of the people charities. An exploratory analysis showed that the slope of a linear regression estimated through the people charity donation points was lower for the group with ASD (m = 0.24) than control group (m = 0.58). A few charities stood out as particularly preferred by the group with ASD. All of these fell into the animal or environment category. Two of these in particular, Canine Assistants and Pineland Preservation Alliance, were remarkable because more than half of the participants with ASD donated to these (whereas most charities only elicited five or six donations from those with ASD).

**Figure 5:**
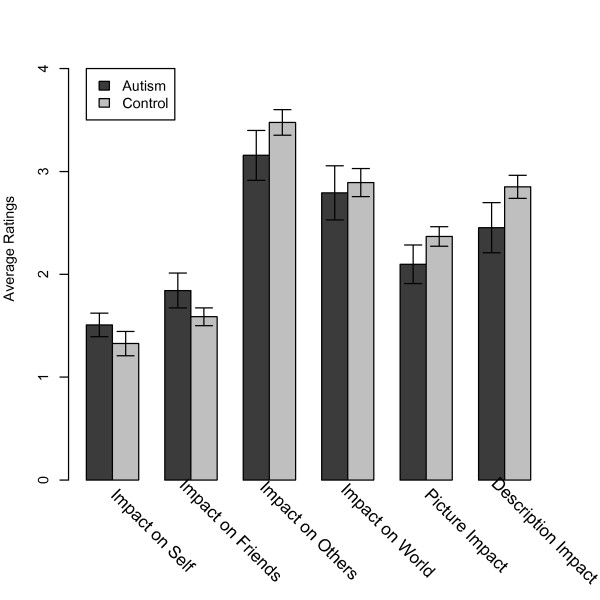
**Ratings given to the charities.** Mean (and SEM) explicit ratings given to the charities, after all donations had been made. See Methods and Appendix 1 for detailed description of the ratings.

**Figure 6:**
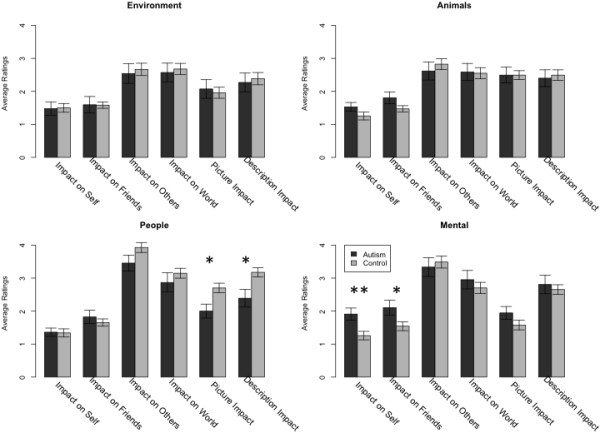
**Ratings broken down by charity category.** Participants with autism spectrum disorders gave significantly lower ratings to the impact of the picture and description just for the people charities. *P < 0.05, **P < 0.01.

Across all charities, both groups generally gave very similar explicit ratings (Figure 5). However, in the people category, the control group gave significantly higher ratings of impact both to the pictures and the narrative associated with the people charities, a pattern not seen for the descriptions of any of the other categories of charities (Figure 6). Specifically, we found a significant group difference for the impact of the picture (ASD: 2.0 versus control 2.7; t(27) = 2.72, *P* < 0.01) and narrative (ASD: 2.4 versus control: 3.2; t(23) = −2.59, *P* <0.02) associated with people charities.

**Figure 7:**
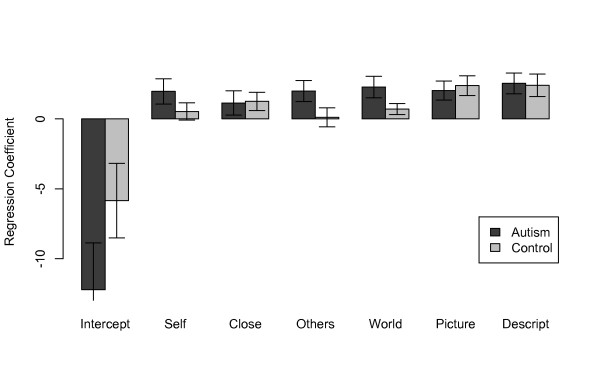
**Regressions: group mean regression coefficients.** We carried out regressions of participants’ ratings onto their donations, individually for each participant. There were no significant differences between groups on any of the regressions.

Regressing ratings onto donation on an individual-by-individual basis resulted in no statistically significant differences between the group means of the regression coefficients (Figure 7). This suggests that while both explicit ratings of the people charities as well as the donations made to them were abnormally low in the group with ASD, the link between evaluations of the descriptions of the charities and donation behavior was unaltered. In the mental health category, participants with ASD gave significantly higher ratings for impact on self (1.9 versus 1.3; t(27) = 2.92, *P* <0.007) and friends (2.1 versus 1.5; t(25) = 2.17, *P* <0.04).

Finally, we carried out exploratory correlations across participants between their mean donation to people charities and several questionnaire-based and diagnostic measures. We did not find any meaningful correlations between mean donation to people charities and age, IQ, income or the perspective taking and empathic concern scale of the IRI. However, there was a negative correlation (r = −0.33) between the ADOS-B subscale (reciprocal social interactions) and mean donation to people charities.

## Discussion

Using a simple charitable donation task, we tested the hypothesis that people with ASD would show reduced social preferences. We found a significant reduction in the frequency and magnitude of donations made to charities benefitting other people compared with those benefitting mental health, animals or the environment. In addition, the group with ASD was less sensitive to specific information that discriminated amongst people charities, donating the same (abnormally low) amount to all of them. Control participants rated the impact of pictures and text descriptions on their donation amount particularly highly for people charities, whereas those with ASD gave significantly lower ratings to their impacts. This suggests that higher donations to people charities may normally be driven by the high social salience that they have, a component that is lacking in people with ASD. Taken together, this pattern of findings supports the hypothesis of abnormal social preferences in ASD and suggests specific reasons for it. The abnormally low ratings of the impact of visual and descriptive information provided for each charity given by the group with ASD argues that socially relevant empathy evoking information was not incorporated into normal valuation for the charity. Consequently, there was little discrimination among the people charities, and the entire category of charities benefitting people was devalued in terms of the actual donations made. While ratings given by people with ASD for the impact of pictures on donations was low for people charities, we did find the group with ASD rated the impact of pictures as high as the control group for animal charities. This is interesting to note because studies have reported people with autism having an easier time connecting with animals than with people.

Several other recent studies have investigated reward processing in people with autism, and have suggested disproportionate impairments in social reward processing, as well as more general impairments in processing rewards across multiple stimulus types. For instance, it was reported that children with autism showed generally impaired implicit reward learning to both money and social stimuli, although the neural response to such stimuli measured with functional magnetic resonance imaging also showed a disproportionate abnormality for the social stimuli in particular [20]. Another study [21] found that the neural response to monetary reward learning was abnormal in people with ASD, but that this abnormality disappeared during processing of interesting objects, possibly corresponding to the restricted interests aspects of the autism phenotype. These studies are broadly consistent with three aspects of our present study: people with ASD donated less overall (a domain-general impairment in reward processing); donated disproportionately less to people charities (a domain-specific impairment in social reward processing); and donated a lot to a few idiosyncratic nonsocial charities (intact or even exaggerated reward processing for a few unusual stimuli). These patterns show that high-functioning people with ASD are not altogether incapable of evaluating stimuli and making reward-based decisions about them - but how they evaluate particular categories of stimuli is abnormal.

Across studies, the specific processes and neural structures that have been found abnormal in reward processing in autism are always a subset of those now well-documented to process the value of stimuli, actions and outcomes in healthy participants. These include regions such as the ventral striatum as well as ventral and medial parts of the prefrontal cortex [22-24], and there is now good evidence that these regions process reward value from all different types of stimuli (such as money, juice or social stimuli), conveyed to these regions through convergent inputs from various sensory association cortices [6-8,15,25-28]. In particular, there is evidence that additional processing is required in order to interpret the value of socially relevant stimuli, originating in part from regions known to process social information, such as cortices in the superior temporal gyrus [12].

Impairments in such additional processing of socially relevant stimuli have been reported in high-functioning people with autism. One study found a remarkably selective impairment in combining outcomes with intentions to evaluate moral actions as good or bad in high-functioning people with autism [29], suggesting that the ability to incorporate multiple sources of social information is particularly compromised. Izuma *et al*. recently reported that people with autism do not show the normal modulation of prosocial behavior (donations to a charity) when they are observed by another person, suggesting that they are insensitive to social reputation effects [30]. In addition, they found that people with autism were insensitive to social reputation effects on charitable donations, and they also observed that overall donations were considerably less than in the control group.

In our present study, we found a similar effect: people with ASD donated less on average, across all stimuli, but in addition they also showed a disproportionate reduction in donations specifically to charities benefitting other people.

One caveat worth mentioning here is while there was no explicit monitoring in our study, as in the Izuma study [30], we concede that participants could have been thinking about the analysis at the end of the experiment and how in principle we could trace who gave to what and how much. This could have created an observer effect that would partly explain the lower average donation amount in people with autism compared with controls.

One special category also worth highlighting is mental health. While we found abnormally low donations in ASD for people charities, we found the highest amount of donations were to the mental health category. When we collapsed the people and mental health categories, the significant difference between participants with ASD and controls disappeared in a one-tailed t-test. An ANOVA comparing non-people (collapsing animal and environment charities) versus people (collapsing people and mental health charities) also showed no significant interaction effects and only a main effect of non-people versus people. This suggests that people with autism treat charities in the mental health category (specifically those benefiting autism) in a special manner, different from their usual donation pattern for other people charities. Indeed the group with ASD gave these charities higher ratings for ‘benefit to self’ and ‘benefit to friends’ than did the control group, as shown in Figure 6. One interpretation of this pattern in the results could be that thinking about charities benefiting people in general requires some empathy. For the control group, this may be one factor driving their donations to the people charities; for the group with ASD, it may be one lacking factor accounting for their low donations to people charities. In the mental health category, however, empathy may not have been required for the participants with ASD to recognize the value since several of these charities were closely related to their own condition.

The phenotype of ASD shows a complex pattern of impairments, typically diagnosed as falling into three classes that together constitute the criteria for clinical diagnosis: language development, reciprocal social interactions, and repetitive behaviors and restricted interests. Arguably, the present findings may contribute to both of the last two, in that they suggest that people with autism have reduced interests in, or preferences for, charities benefitting people as compared to charities benefitting other categories. Moreover, we found a few charities that elicited unusually high donations from the group with ASD, a finding that should be followed up in future studies to better understand what it is about these particular charities that makes them preferable to people with autism. It is also interesting that we found a negative correlation between the amounts that participants with ASD donated to the people charities and the ADOS-B subscale. This subscale comprises items assessing unusual eye contact, facial expression directed to others, empathy and comments on others’ emotions, responsibility, quality of social overtures, quality of social response and amount of reciprocal social communication. While exploratory, this finding provides preliminary evidence that the abnormal social preferences revealed in our task may relate to abnormal social interactions in people with autism.

Returning to the social motivation hypothesis of autism [3,9,10], it remains an intriguing question how precisely the pattern of impairments we report here emerges during development. One possibility is that early domain-general impairments in reward processing, in a developmental context, give rise to impairments disproportionate for social stimuli [31]. Similarly, early domain-general impairments in integrating complex contextual information may result in impairments particularly acute for social stimuli, simply because these draw more upon integrating multiple sources of information. An important future task will be to map out the abilities, and the concomitant brain responses, of people with ASD to process and evaluate a broad range of stimuli. Finally, a full understanding of motivated behavior in ASD will also need to examine the flip-side of reward processing: aversive behavior elicited by actively disliked stimuli.

## Conclusions

Using a simple charitable donation task, we found a significant reduction in the frequency and magnitude of donations made by people with autism to charities benefitting people compared to other charity categories. In addition, the group with ASD was less sensitive to specific information that discriminated amongst people charities, donating the same (abnormally low) amount to all of them. Whereas the control group rated the impact of pictures and text descriptions on their donation amount as particularly high for people charities, the group with ASD gave significantly lower ratings to these. This suggests that higher donations to people charities may normally be driven by the high social salience that they have, a component that is lacking in ASD. Taken together, this pattern of findings supports the hypothesis of abnormal social preferences in ASD and suggests specific reasons for it. The abnormally low ratings of the impact of visual and descriptive information provided for each charity given by the participants with ASD argues that socially relevant empathy evoking information may not have been incorporated into normal valuation for the charity. These findings provide evidence for a domain-specific impairment in social cognition in ASD, and in particular in linking otherwise intact social knowledge to the construction of value signals on which preferences regarding other people are based.

## Appendix 1: complete list of questions (ratings) asked

· How much do you think a $500,000 donation to this charity would help you personally?

· How much do you think a $500,000 donation to this charity would help others you care about?

· How much do you think a $500,000 donation to this charity would help other people?

· How much do you think a $500,000 donation to this charity would help the world?

· To what extent does the charity picture increase your willingness to give?

· To what extent does the charity description increase your willingness to give?

## Competing interests

The authors declare that they have no competing interests.

## Authors’ contributions

AL, KT, AR and RA designed research; AL and KT performed research; AL analyzed data; and AL and RA wrote the paper. All authors read and approved the final manuscript.

## Supplementary Material

Additional file 1 **Table S1.** Complete list of charities. Click here for file

## References

[B1] Klin A Danovitch JH Merz AB Volkmar F Circumscribed interests in higher-functioning individuals with autism spectrum disorder: an exploratory study Res Pract Persons Severe Disabl 2007 32 89 100

[B2] Sasson N Turner-Brown LM Holtzclaw T Lam KS Bodfish J Children with autism demonstrate circumscribed attention during passive viewing of complex social and nonsocial picture arrays Autism Res 2008 1 31 42 10.1002/aur.4 19360648 PMC3709846

[B3] Dawson G Meltzoff AN Osterling J Rinaldi J Brown E Children with autism fail to orient to naturally occurring social stimuli J Autism Dev Disord 1998 28 479 485 10.1023/A:1026043926488 9932234

[B4] Baron-Cohen S Mindblindness: An Essay on Autism and Theory of Mind 1997 MIT Press, Cambridge

[B5] Frith U Mind blindness and the brain in autism Neuron 2001 32 969 979 10.1016/S0896-6273(01)00552-9 11754830

[B6] Chib VS Rangel A Shimojo S O'Doherty JP Evidence for a common representation of decision values for dissimilar goods in human ventromedial prefrontal cortex J Neurosci 2009 29 12315 12320 10.1523/JNEUROSCI.2575-09.2009 19793990 PMC6666137

[B7] Lin A Adolphs R Rangel A Social and monetary reward engage overlapping neural substrates Soc Cogn Affect Neurosci 2012 7 274 281 10.1093/scan/nsr006 21427193 PMC3304477

[B8] O’Doherty J Winston J Critchley H Perrett D Burt DM Dolan RJ Beauty in a smile: the role of medial orbitofrontal cortex in facial attractiveness Neuropsychologia 2003 41 147 155 10.1016/S0028-3932(02)00145-8 12459213

[B9] Dawson G Carver L Meltzoff AN Panagiotides H McPartland J Webb B Neural correlates of face and object recognition in young children with autism spectrum disorder, developmental delay, and typical development Child Dev 2002 73 700 717 10.1111/1467-8624.00433 12038546 PMC3651041

[B10] Grelotti DJ Gauthier I Schultz RT Social interest and the development of cortical face specialization: what autism teaches us about face processing Dev Psychobiol 2002 40 213 225 10.1002/dev.10028 11891634

[B11] Grelotti DJ Klin A Gauthier I Skudlarski P Cohen DJ Gore JC Volkmar FR Schultz RT fMRI activation of the fusiform gyrus and amygdala to cartoon characters but not to faces in a boy with autism Neuropsychologia 2005 43 373 385 10.1016/j.neuropsychologia.2004.06.015 15707614

[B12] Hare TA Camerer CF Knoepfle DT O'Doherty JP Rangel A Value computations in ventral medial prefrontal cortex during charitable decision making incorporate input from regions involved in social cognition J Neurosci 2010 30 583 590 10.1523/JNEUROSCI.4089-09.2010 20071521 PMC6633003

[B13] Frith CD The social brain? Philos Trans R Soc Lond B Biol Sci 2007 362 671 678 10.1098/rstb.2006.2003 17255010 PMC1919402

[B14] Singer T Seymour B O'Doherty J Kaube H Dolan RJ Frith CD Empathy for pain involves the affective but not sensory components of pain Science 2004 303 1157 1162 10.1126/science.1093535 14976305

[B15] Harris A Adolphs R Camerer CF Rangel A Dynamic construction of stimulus values in the ventromedial prefrontal cortex PLoS One 2011 6 e21074 10.1371/journal.pone.0021074 21695081 PMC3114863

[B16] Lord C Risi S Lambrecht L Cook EH Leventhal BL DiLavore PC Pickles A Rutter M The autism diagnostic observation schedule-generic: a standard measure of social and communication deficits associated with the spectrum of autism J Autism Dev Disord 2000 30 205 223 10.1023/A:1005592401947 11055457

[B17] Lord C Rutter M Le Couteur A Autism Diagnostic Interview - Revised: a revised version of a diagnostic interview for caregivers of individuals with possible pervasive developmental disorders J Autism Dev Disord 1994 24 659 685 10.1007/BF02172145 7814313

[B18] Wechsler DA The Wechsler Adult Intelligence Scale - Revised 1981 The Psychological Corporation, New York

[B19] Davis MH Measuring individual differences in empathy: Evidence for a multidimensional approach J Pers Soc Psychol 1983 44 113 126

[B20] Scott-Van Zeeland A Dapretto M Ghahremani DG Poldrack RA Bookheimer SY Reward processing in autism Autism Res 2010 3 53 67 20437601 10.1002/aur.122 PMC3076289

[B21] Dichter GS Felder JN Green SR Rittenberg AM Sasson NJ Bodfish JW Reward Circuitry Function in Autism Spectrum Disorders Social, Cognitive, Affective Neurosci 2012 7 160 172 10.1093/scan/nsq095 PMC3277365 21148176

[B22] Rangel A Camerer C Montague PR A framework for studying the neurobiology of value-based decision making Nat Rev Neurosci 2008 9 545 556 10.1038/nrn2357 18545266 PMC4332708

[B23] Rangel A Hare T Neural computations associated with goal-directed choice Curr Opin Neurobiol 2010 20 262 270 10.1016/j.conb.2010.03.001 20338744

[B24] Wallis JD Orbitofrontal cortex and its contribution to decision-making Annu Rev Neurosci 2007 30 31 56 10.1146/annurev.neuro.30.051606.094334 17417936

[B25] Grabenhorst F D’Souza AA Parris BA Rolls ET Passingham RE A common neural scale for the subjective pleasantness of different primary rewards NeuroImage 2010 51 1265 1274 10.1016/j.neuroimage.2010.03.043 20332031

[B26] Izuma K Saito DN Sadato N Processing of social and monetary rewards in the human striatum Neuron 2008 58 284 294 10.1016/j.neuron.2008.03.020 18439412

[B27] Janowski V Camerer C Rangel A Empathic choice involves vmPFC value signals that are modulated by social processing implemented in IPL Soc Cogn Affect Neurosci 2012 [Epub ahead of print] 10.1093/scan/nsr086 PMC3575723 22349798

[B28] O’Doherty JP Deichmann R Critchley H Dolan RJ Neural responses during anticipation of a primary taste reward Neuron 2002 33 815 826 10.1016/S0896-6273(02)00603-7 11879657

[B29] Moran JM Young L Saxe R Lee SM O'Young D Mavros P Gabrieli J Impaired theory of mind for moral judgement in high-functioning autism PNAS 2011 108 2688 2692 10.1073/pnas.1011734108 21282628 PMC3041087

[B30] Izuma K Matsumoto K Camerer C Adolphs R Insensitivity to social reputation in autism PNAS 2011 108 17302 17307 10.1073/pnas.1107038108 21987799 PMC3198313

[B31] Triesch J Teuscher C Deak GO Carlson E Gaze following: why (not) learn it? Dev Sci 2006 9 125 147 10.1111/j.1467-7687.2006.00470.x 16472311

