# Supplementary material for: Reduced social preferences in autism: evidence from charitable donations
Source: J Neurodev Disord. 2012 May 17;4(1):8. doi: 10.1186/1866-1955-4-8 (PMC3436698; doi:10.1186/1866-1955-4-8)
Supplement: Additional file 1 — Table S1. Complete list of charities. [file 1866-1955-4-8-S1.doc]

Table S1: complete list of charities

| **Charity Name** | **Category** |
| --- | --- |
| Achievement Centers for Children | mental health |
| African Wildlife Foundation | animal |
| American Bird Conservancy | animal |
| American Kennel Club | animal |
| American Red Cross | people |
| Animal Haven | animal |
| Animal Rescue | animal |
| Anxiety Disorders Association of America | mental health |
| Autism Research Institute | mental health |
| Blue Card | people |
| Brain Tumor Society | people |
| Camphill Village Kimberton Hills | mental health |
| Canine Assistants | animal |
| CARE | people |
| Chicago Foundation for Women | people |
| Child Abuse Prevention Center | people |
| Direct Relief International | people |
| Dogs for the Deaf | animal |
| Fisher Center for Alzheimer's Research Foundation | people |
| Global Fund for Women | people |
| Heal the Bay | environmental |
| Horizons for Homeless Children | people |
| Infant Crisis Services | people |
| International Eye Foundation | people |
| International Rett Syndrome Foundation | mental health |
| Make-A-Wish Foundation International | people |
| Mercy Medical Airlift | people |
| National Breast Cancer Foundation | people |
| National Center for Missing and Exploited Children | people |
| National Childhood Cancer Foundation | people |
| National Inclusion Project | mental health |
| National Ovarian Cancer Coalition | people |
| National Spinal Cord Injury Association | people |
| National Transplant Assistance Fund | people |
| Oprah's Angel Network | people |
| Organization for Autism Research | mental |
| Parkinson's Disease Foundation | people |
| Pasadena Humane Society and SPCA | animal |
| Pine Tree Society | mental health |
| Pinelands Preservation Alliance | environmental |
| Save the Children | people |
| Society for the Protection of New Hampshire Forests | environmental |
| Southeast Alaska Conservation Council | environmental |
| Spinal Bifida Association | people |
| The American Chestnut Foundation | environmental |
| The Children's Clinic | people |
| The Churchill School and Center | mental health |
| The WILD Foundation | animal |
| Thoroughbred Retirement Foundation | animal |
| Upper Raritan Watershed Association | environmental |
